# Coro2b, a podocyte protein downregulated in human diabetic nephropathy, is involved in the development of protamine sulphate-induced foot process effacement

**DOI:** 10.1038/s41598-019-45303-y

**Published:** 2019-06-20

**Authors:** Angelina Schwarz, Katja Möller-Hackbarth, Lwaki Ebarasi, David Unnersjö Jess, Sonia Zambrano, Hans Blom, Annika Wernerson, Mark Lal, Jaakko Patrakka

**Affiliations:** 1 Karolinska Institutet/AstraZeneca Integrated Cardio Metabolic Centre, Department of Laboratory Medicine, Karolinska Institutet at Karolinska University Hospital Huddinge, Stockholm, Sweden; 2 Science for Life Laboratory, Department of Applied Physics, Royal Institute of Technology, Solna, Sweden; 3 Division of Renal Medicine, Department of Clinical Sciences, Intervention and Technology, Karolinska Institutet, Stockholm, Sweden; 4 Bioscience, Cardiovascular, Renal and Metabolism, Innovative Medicines Biotech Unit, AstraZeneca, Gothenburg, Sweden

**Keywords:** Actin, Diabetic nephropathy, IgA nephropathy, Podocytes

## Abstract

Podocytes have an important role in the pathogenesis of diabetic nephropathy (DN). Podocyte foot process effacement, mediated largely by the actin-based cytoskeleton of foot processes, is commonly detected in DN and is believed to be a key pathogenic event in the development of proteinuria. In this study, we identified coronin 2b (Coro2b), a member of known actin-regulating proteins, the coronins, as a highly podocyte-enriched molecule located at the cytoplasmic side of the apical plasma membrane. Studies in human renal biopsies show that glomerular Coro2b expression is significantly down-regulated in patients with DN. Studies in knockout mice indicate that Coro2b is not required for the development or maintenance of the glomerular filtration barrier. Moreover, inactivation of Coro2b specifically in podocytes does not affect the outcome of nephropathy in a streptozotocin-induced diabetes model. However, Coro2b seems to modulate the reorganization of foot processes under pathological conditions as Coro2b knockout podocytes are partially protected from protamine sulfate perfusion-induced foot process effacement. Taken together, our study suggests a role for Coro2b in the pathogenesis of glomerulopathies. Further studies regarding the involvement of Coro2b in podocyte health and diseases are warranted.

## Introduction

Diabetic nephropathy (DN) is a major cause of end stage renal disease and associated with a significantly increased risk for cardiovascular diseases^1,2^. The incidence of type 2 diabetes (T2DM) is rising worldwide and no effective new treatment options for DN have been developed since the introduction of renin-angiotensin-aldesterone-system (RAAS) blockade^3^. Pharmaceutical development has been hampered by our poor understanding of causative molecular mechanisms driving disease progression in kidney tissue.

DN is primarily affecting renal glomeruli and glomerular podocytes are believed to play a central role in disease evolution. Podocytes are highly specialized epithelial cells that surround glomerular capillaries with their foot processes and form the final barrier for renal filtration. A hallmark sign of podocyte injury in DN and other proteinuric disorders is the effacement of foot processes. This change is regulated by the reorganization of the actin cytoskeleton. The maintenance and dynamic changes of the foot process actin cytoskeleton involves a complex interplay between actin and a number of actin-binding proteins, such as synaptopodin and alpha-4-actinin^4^. The central role of the foot process actin cytoskeleton is highlighted by studies showing that mutations in actin-associated podocyte proteins are the cause of inherited proteinuric diseases in man^5^. Interestingly, it has been reported that pharmaceutical targeting of the actin cytoskeleton can attenuate progression of chronic kidney disease in mice^6^.

In this study, we identified coronin 2b (Coro2b), a member of known actin-regulating proteins the coronins, as a highly podocyte enriched protein down-regulated in DN^7,8^. Our results suggest that Coro2b modulates actin cytoskeleton dynamics in foot processes under pathological conditions as podocyte specific Coro2b knockout mice are partially protected from the development of foot process effacement after injury.

## Results

### Coro2b is highly enriched in glomerular podocytes

We have previously analyzed the mouse glomerular transcriptome in detail using microarrays^8^. One of the highly glomerulus-enriched transcripts identified encoded for a member of the coronin family of actin regulators, Coro2b. Investigation of the Human Protein Atlas database (www.proteinatlas.org) showed a highly specific expression of Coro2b protein in the human glomerulus in comparison to the surrounding tubular tissue. Therefore, we studied the expression of Coro2b in the human kidney cortex in more detail.

**Figure 1:**
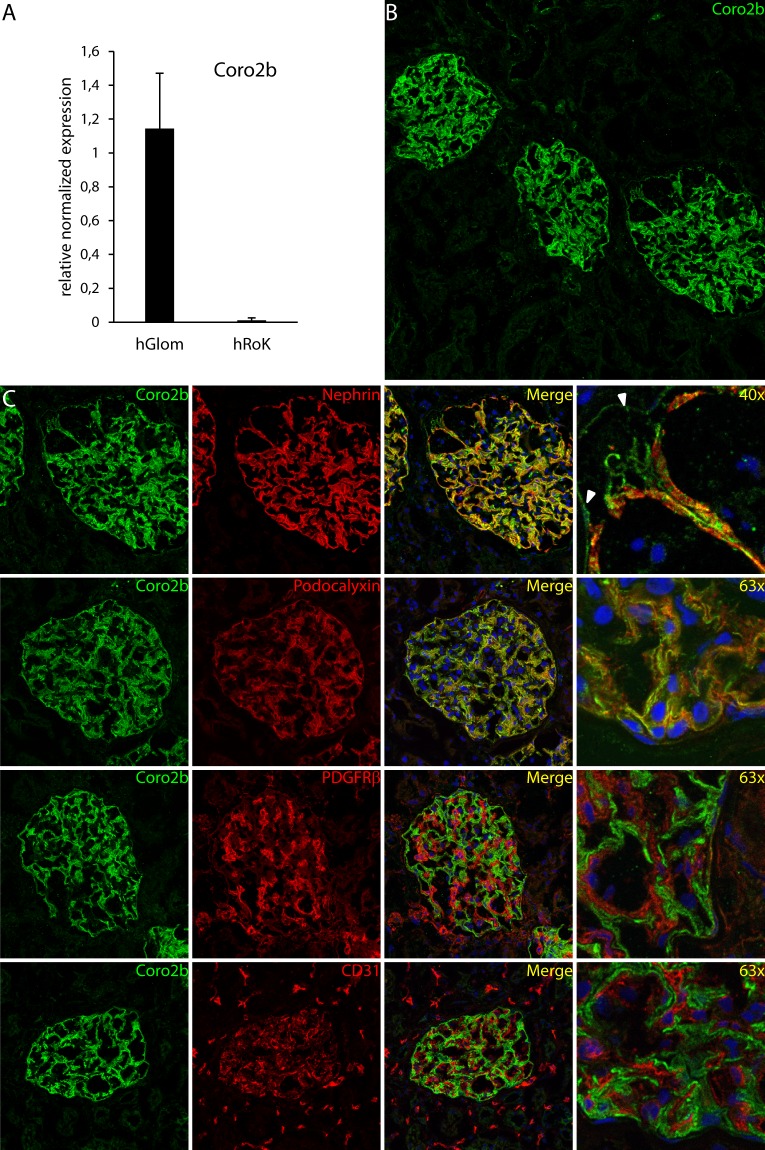
Expression of Coro2b in the kidney cortex. (**A**) qPCR with human cDNA from isolated glomeruli (hGlom) and rest of kidney fraction (hRoK) shows that Coro2b is highly enriched in glomeruli. Relative expression was normalized to 28S with the 2^−ΔΔct^ formula, two tailed distributed t-test: p < 0.001. (**B**) Immunofluorescence staining of human kidney tissue shows that Coro2b expression is restricted to glomeruli within the cortex. (**C**) Immunofluorescence co-stainings of Coro2b (green) with glomerular cell markers (red) nephrin (podocyte), podocalyxin (podocyte), PDGFRβ (mesangial) and CD31 (endothelial) show that Coro2b is expressed in the human podocyte. Signal overlap occurs only with foot process proteins nephrin and podocalyxin (yellow). Double labeling of Coro2b with nephrin unravels apical podocyte staining for Coro2b (arrow). Magnifications: B - 20x, C - 40x and 63x.

In human isolated kidney fractions, qPCR showed a strong expression of Coro2b transcript in the glomerulus and very low to null expression in the rest of kidney fraction (Fig. 1A). Expression at the protein level was assessed using immunofluorescence staining of adult human kidney tissue. A strong signal was detected within glomerular tufts and no signal was observed in the surrounding tissue (Fig. 1B). Double staining with the podocyte foot process marker nephrin showed overlapping reactivity with Coro2b (Fig. 1C). However, Coro2b was also detected on the apical side of nephrin staining. Double labeling with apical plasma membrane marker podocalyxin showed more complete overlapping signal, suggesting that Coro2b was localized at the cytoplasmic side of the apical plasma membrane. In contrast to this, co-labeling with the endothelial marker CD31 and the mesangial marker platelet-derived growth factor beta (PDGFRβ) exhibited no significant co-localization (Fig. 1C). The specificity of the staining pattern was validated using a second anti-Coro2b antibody targeting a different epitope of the protein. This antibody gave a very similar staining pattern in glomerular podocytes (Suppl. Fig. 1). The results indicate that the expression of Coro2b in kidney tissue is highly enriched in podocytes.

### Coro2b localizes to the apical plasma membrane of podocytes

**Figure 2:**
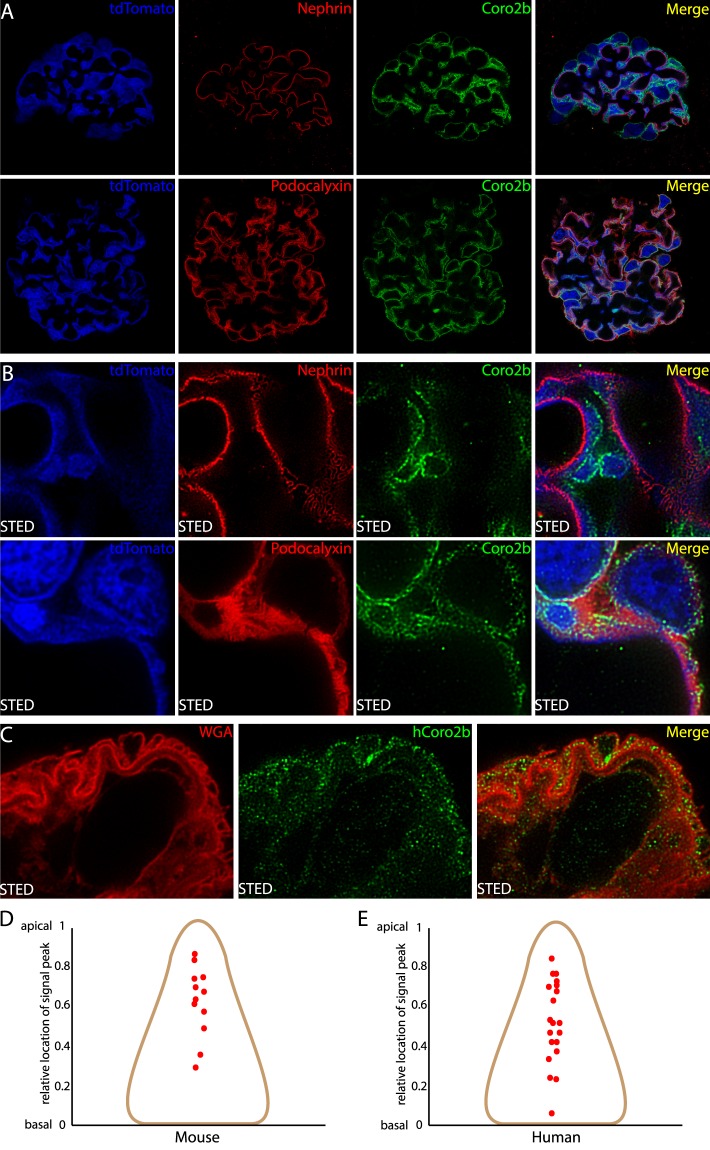
Localization of Coro2b in human and mouse podocyte foot processes. (**A**,**B**) Immunofluorescent staining for Coro2b (green) and nephrin (red) or podocalyxin (red) in a transgenic tdtomato (blue) expressing podocyte mouse line imaged with confocal and stimulated emission depletion (STED) microscopy. Coro2b is detected in podocytes without any significant co-localization with nephrin, whereas overlapping signal with podocalyxin is observed. (**C**) Co-staining of Coro2b with epithelial cell membrane binding wheat germ agglutinin (WGA) in human podocytes, captured with STED microscopy. Coro2b is detected on the apical side of podocyte foot processes (**D**,**E**) Localization of Coro2b signal in mouse and human foot processes as detected by STED microscopy. The y-axis represents the relative localization of signal peak between the basal and apical membranes. Coro2b is dominantly detected at the apical side of mouse and human foot processes. Images were deconvoluted using SVI Huygen’s software (Hilversum, the Netherlands). Magnifications, A - 100x, B, C - 100x STED.

As Coro2b was recently reported to localize to focal adhesions at the basal aspects of podocyte foot processes^9,10^, we analyzed in more detail the distribution of Coro2b in podocytes. We applied an optical clearing protocol to a mouse kidney expressing tdtomato in podocytes to perform high resolution imaging. Immunofluorescence staining in this tissue showed almost no co-localization between nephrin and Coro2b, whereas podocalyxin showed overlapping reactivity with Coro2b (Fig. 2A). Next, we used super-resolution stimulated emission depletion (STED) microscopy in combination with optical clearing and deconvolution, which allows to visualize the intricate foot process structures. This analysis unraveled Coro2b staining mostly at the apical aspects of podocytes as shown by double labeling with nephrin and podocalyxin (Fig. 2B). We used the same optical clearing and STED microscopy for human kidney tissue. Foot processes in human kidneys were visualized by wheat germ agglutinin (WGA), which binds sialic acid and *N*-acetylglucosaminyl residues in epithelial cell membranes. Like in mouse, we detected Coro2b in human podocytes mostly at the apical side of foot processes (Fig. 2C). Finally, we determined the localization of Coro2b in foot processes by creating line plots from basal to apical membrane and by measuring the localization of the peak of Coro2b signal. This analysis showed that Coro2b mainly localizes at the apical side of podocytes and almost no signal at the basal side was detected (Fig. 2D,E). The results indicate that Coro2b in podocytes is localized at the cytoplasmic side of the apical plasma membrane.

### Coro2b expression is down-regulated in DN but not in IgA nephropathy (IgAN) or membranous nephropathy (MN)

**Figure 3:**
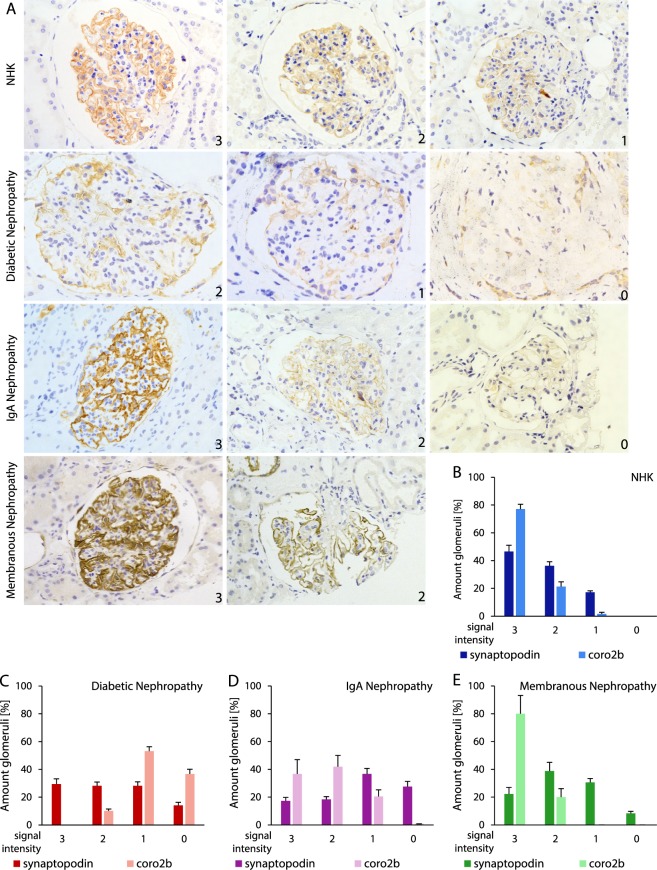
Differential expression of Coro2b in DN, IgA nephropathy and membranous nephropathy. (**A**) Immunohistochemistry for Coro2b in normal human kidney (NHK), diabetic nephropathy (DN), IgA nephropathy (IgAN) and membranous nephropathy (MN). Immunoreactivity is detected as a linear line around glomerular capillary loops in all samples. The staining is weaker in DN but not in IgAN or MN when compared to NHK. For semi-quantitative scoring following signal intensities were used: 3 = strong, 2 = medium, 1 = low and 0 = zero. (**B–E**) Staining intensities for Coro2b and synaptopodin as shown by average and STDEV in control and disease samples. With a two way ANOVA the correlation/interaction between Coro2b and synaptopodin was calculated for each disease group: (**B**) NHK p = 0.00016, (**C**) DN p = 0.0002, (**D**) IgAN p = 0.078, (**E**) MN p = 4.19E-06. Magnifications: A - x20.

To analyze the role of Coro2b in acquired human glomerular diseases, we studied the expression pattern of Coro2b using immunoperoxidase staining in renal biopsies collected from patients with DN, IgA nephropathy (IgAN) and membranous nephropathy (MN). Clinical parameters of the patients are found in supplemental tables 1 and 2. By immunohistochemistry, no obvious re-distribution of Coro2b was detected as the staining showed a linear line around capillary loops in both control and disease samples (Fig. 3A). However, we could detect a clear reduction of signal intensity in patients with DN. We validated the expression change through a semi-quantitative scoring, in which the glomerular staining intensity was graded from 3 to 0 in each glomerulus, with 3 representing the strongest staining intensity and 0 representing no visible staining. In control samples, 77% of glomeruli were graded as “strong signal” (3) for Coro2b (Fig. 3B). In contrast to this, none of the glomeruli in DN showed “strong signal” for Coro2b and 90% of DN glomeruli exhibited only low/undetectable staining (1(53%) & 0(37%)), indicating a decreased expression of Coro2b (Fig. 3C). Interestingly, we did not observe similar down-regulation of Coro2b expression in patients with IgAN or MN. In IgAN, 37% of glomeruli showed “strong signal” (3) and only 1% were graded as 0, whereas in MN 80% showed “strong signal” (3) and none were graded as 0 (Fig. 3D,E). In contrast to Coro2b, staining for synaptopodin, another podocyte specific protein, showed only modest reduction in all three glomerulopathies. To analyze the expression of Coro2b at the mRNA level in DN, we extracted microarray data generated from human isolated glomeruli previously by Woroniecka *et al*.^7^. In this data, Coro2b was found to be among the top 5 down-regulated genes in DN glomeruli compared to control glomeruli (Suppl. Fig. 2). Taken together, the expression of Coro2b is down-regulated in DN glomeruli, suggesting that it might have a pathogenic role in this disease.

### Generation and characterization of Coro2b knock-out mouse line

**Figure 4:**
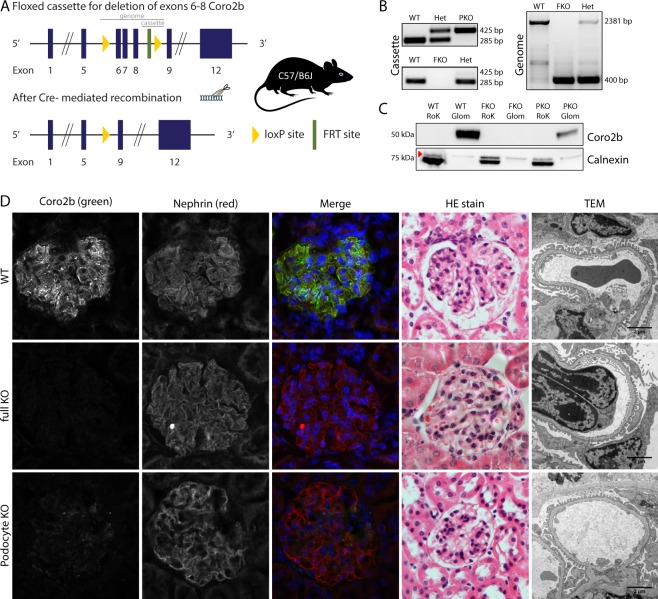
Generation and characterization of Coro2b KO mouse line. (**A**) Genetic targeting scheme for the deletion of exons 6–8 of Coro2b in mice. LoxP sites allow the generation of podocyte-specific or full body KO animals. (**B**) Genotyping strategy amplifies for the floxed allele (location indicated in **A**) a 425 bp and for the WT allele a 285 bp product. For the full KO allele, designed primers (location indicated in **A**) generate a 400 bp and for the WT allele 2381 bp product. (**C**) Western blot of isolated glomeruli (Glom) and tubular (RoK) fractions from control, full body KO (FKO) and podocyte-specific KO (PKO) show that Coro2b is in mice only expressed in the glomeruli and the successful ablation of the protein in the KOs. Calnexin was used as loading control (the band for calnexin is indicated by the red arrow). (**D**) Histological characterization of 4–6 month old KO mice. Immunofluorescent staining shows a total loss of signal in FKO glomeruli and a significant reduction in PKOs. Hematoxylin and eosin-staining and standard transmission electron microscopic evaluation showed no changes between the three groups. Magnifications: 40x and 11500x. B + C Un-cropped pictures are provided in supplementary.

Next, we wanted to analyze the physiological function of Coro2b *in vivo* by generating a knock-out (KO) mouse line. We targeted the exons 6 to 8 out of total 12 exons with loxP sites, and crossed this transgene with Ella-cre^11^ and podocin-cre lines^12^ in order to generate global and podocyte-specific KO animals, respectively (Fig. 4A). Genotyping strategy for the constitutive KO generated a 400 bp and the wild type allele a 2381 bp product, whereas the conditional allele amplified a 425 bp and the corresponding wild type allele a 285 bp band (Fig. 4B).

The targeting strategy resulted in ablation of functional Coro2b protein as we detected no Coro2b band in Western blotting of the constitutive KO kidney tissue and reduced protein level in conditional KO tissue (Fig. 4C). In line with this, immunofluorescence staining with Coro2b antibody showed a total loss of signal in glomeruli of constitutive KO animals and a clear reduction of signal in conditional KO mice (Fig. 4D). To visualize the glomeruli, a specific antibody for nephrin has been used. Constitutive KO mice were born in a normal Mendelian distribution and grew up without exhibiting any notable differences compared to their wildtype littermates (data not shown). No major organ showed any obvious macroscopical or histological alterations (data not shown). Renal glomerular histology was indistinguishable from control animals as observed at 4 to 6 month of age (Fig. 4D). Electron microscopic examination showed well developed podocyte foot processes connected by intact slit diaphragms (Fig. 4D). Moreover, no significant albuminuria was detected in constitutive or conditional Coro2b KO animals (data not shown).

### Absence of Coro2b in podocytes does not affect response to hyperglycemic conditions in mouse

**Figure 5:**
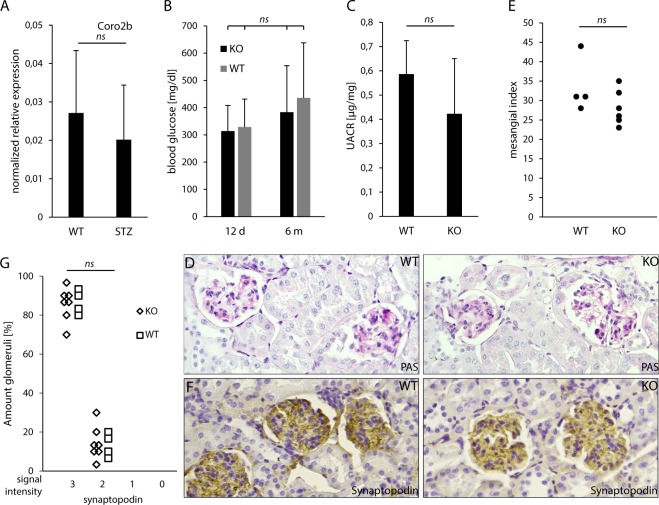
Inactivation of Coro2b in podocytes does not affect the outcome of nephropathy in STZ-induced diabetes model. (**A**) qPCR for Coro2b does not show significant differential expression between healthy wild type and STZ-induced diabetic glomeruli. Relative expression was normalized to GAPDH with the 2^−ΔΔct^ formula, two tailed distributed t-test: p = 0.54. (**B**) Blood glucose measurement 13 days and 6 months after the induction of diabetes shows that all animals are hyperglycemic and both groups show similar plasma glucose levels. One way ANOVA: p = 0.56. (**C**) Measured albumin/creatinine ratio (UACR) shows that both groups of STZ treated KO and control animals were not albuminuric at 5 months. Two tailed distributed t-test: p = 0.24. (**D**) Six months after the induction of diabetes, histological examination shows glomerular damage reminiscent of human DN, including mesangial matrix expansion. (**E**) Quantification of mesangial expansion in diabetic control and KO animals shows no significant difference between the two groups. Each dot represents the sum of 20 randomly counted glomeruli per animal, which were graded for their sclerotic severity. Two tailed distributed t-test: p = 0.2. (**F**,**G**) Synaptopodin staining and respective semi-quantitative scoring showed no significant differences between KO and litter-mate control animals. Two tailed distributed t-test: p = 0.98. Magnifications: C - x20.

As Coro2b expression was downregulated in patients with DN, we challenged conditional podocyte Coro2b KO mice with hyperglycemic conditions. We induced diabetes in 7 KO and 4 litter-mate control animals at 8 weeks of age using streptozotocin (STZ). We detected a trend for down-regulation of Coro2b expression in glomeruli after induction of diabetes in wild type mice but this was not statistically significant (Fig. 5A). All animals showed increased blood glucose values, which were similar in both KO and control mice (Fig. 5B). No significant differences were detected in albuminuria levels (Fig. 5C). The experiment was terminated 6 months after the injection of STZ. Histological examination of diabetic animals showed that both KO and control animals developed mesangial matrix expansion and glomerulosclerosis reminiscent of DN (Fig. 5D). Semi-quantitative scoring of glomerular changes showed no difference between KO and litter-mate control animals (Fig. 5E). To evaluate possible podocyte damage, we stained kidneys for synaptopodin. Semi-quantitative scoring of synaptopodin staining revealed no significant differences between KO and litter-mate control animals (Fig. 5F,G). Taken together, absence of Coro2b in podocytes did not affect the outcome of nephropathy in a STZ-induced model of diabetes.

### Absence of Coro2b in podocytes modulates structure of foot processes in a podocyte targeting mouse model

**Figure 6:**
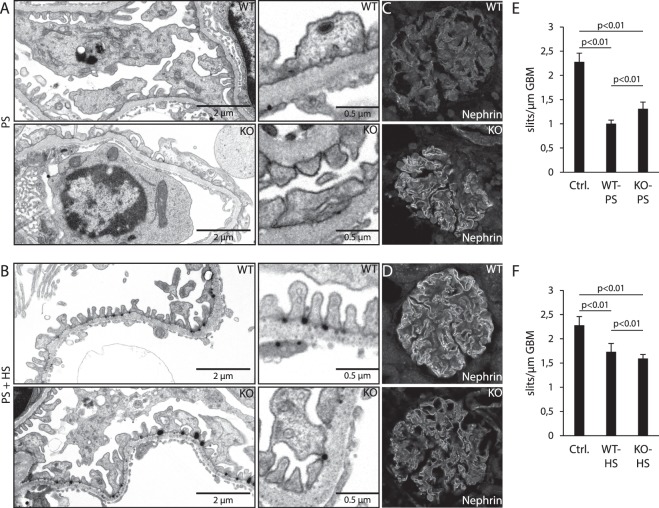
Coro2b-deficiency in podocytes partially protects against protamine sulfate induced foot process effacement. (**A**,**B**) Perfusion with protamine sulfate (PS) induced foot process effacement, which could be rescued by heparin sulfate (HS) perfusion in both KO and littermate control animals as detected by transmission electron microscopy. (**C**) Immunofluorescent signal for nephrin in PS perfused glomeruli shows a more granular staining pattern in WT than KO glomeruli. (**D**) Immunofluorescent signal for nephrin in glomeruli rescued with HS perfusion shows sharper signal in WT than KO glomeruli. **E** KO glomeruli showed significantly less effacement as calculated by number of slits per GBM length. **F** Perfusion with heparin sulfate can partially reverse the effects of protamine sulfate on foot processes. Statistics: One way ANOVA with Tukey-Kramer post-hoc correction analysis gives for all interactions a p < 0.01. Magnification: A, B - 11500x and C, D - 40x.

As Coro2b has been shown to associate with the F-actin cytoskeleton in neurite tips^10^, we decided to use a kidney specific stress model in which the actin cytoskeleton in podocyte foot processes goes through dynamic reorganization induced by perfusion with protamine sulfate (PS) followed by perfusion with heparin sulfate (HS). PS alters the anionic charge in the glomerulus, affecting the actin cytoskeleton and leading to foot process effacement, whereas HS binds to PS and reverses the effect in the podocytes^13^. A total of 17 mice, 9 conditional KOs and 8 litter-mate controls, were perfused with PS only (KO n = 5; WT n = 4) or with PS followed by HS perfusion (KO n = 4; WT n = 4). Electron microscopic analysis validated the induction of foot process effacement in animals perfused with PS and the partial reverse of the phenotype by HS (Fig. 6A,B). The measurement of foot process effacement determined by calculating the frequency of slit diaphragms per glomerular basement membrane (GBM) length showed that KO animals were significantly protected from the development of foot process effacement (Fig. 6E). In addition, there was a significant difference in the recovery of foot process architecture between groups (Fig. 6F). To get more insights, we analyzed the staining pattern of nephrin in the perfused kidneys. It appeared that the WT glomeruli generally showed more granular staining pattern after the perfusion with PS when compared to KO glomeruli (Fig. 6C). Similarly, it appeared that the staining for nephrin was sharper in WT glomeruli than in KO glomeruli after rescue with HS perfusion (Fig. 6D). Taken together, the results suggest that Coro2b modulates the response to both PS and HS treatment during the development and rescue of foot process effacement.

## Material and Methods

### Human kidney tissues

Control kidneys were obtained from patients who underwent nephrectomy due to renal tumors, whereas renal biopsies were from patients with DN, IgAN and MN at Karolinska University Hospital (Stockholm, Sweden), in accordance with the recommendations of the regional Ethical Committee (Regionala Etikprövningsämnden i Stockholm 2010/579-31; 2017/58-31/4). All samples were collected from patients who gave informed consent to study participation. Glomeruli were isolated from nephrectomized kidneys using a modified sieving method^14^. First, the cortex tissue was briefly washed in phosphate-buffered-saline (PBS) and capsule remains were removed, followed by mincing of the tissue. The homogenous cortex fraction was then sieved with a mesh size of 500 µm followed by sieving with a 250 µm mesh size. The eluate was loaded onto the last sieve of 150 µm mesh size and washed with PBS to remove all tubular tissue. Glomeruli were collected from the sieve surface and the flow-through was collected as tubular fraction. Tissue isolates were immersed either in TRIzol™ Reagent (Thermo Fisher Scientific, Germany) (for cDNA collection) or RIPA-buffer (for protein lysates).

### PCR experiments

Mouse glomeruli were isolated according to previously described method^15^ and PCR experiments were run using standard protocols. The primers used are listed in supplemental table 3. qPCR experiments were normalized to 28S and evaluated using the ΔΔC_t_ – method to measure relative mRNA levels.

### Western blotting, immunohistochemistry staining and electron microscopy

Western blots and immunohistochemistry were performed according to previously described methods^16^. STED microscopy and optical clearing was carried out according to previously published protocol^17^, with the following modifications: Kidneys were pre-fixed in 4% PFA in 1X PBS at 4 °C overnight and then transferred to monomer solution (4% acrylamide, 0.25% VA-044 thermal initiator in 1X phosphate-buffered saline) at 4 °C overnight, then polymerized for 3 h at 37 °C and cleared in clearing solution for 2 days before immunostaining, mounting and imaging. Electron microscopy was performed using standard protocols, including fixation with 2.5% glutaraldehyde by the EM facility of the Karolinska University Hospital. The list of used antibodies is shown in supplemental table 4.

### Scoring and detection methods

The line plots to detect protein localization from the STED images were created using ImageJ and Matlab. Line ROI’s (region of interest) of appropriate thickness were drawn from basal to apical part of FP’s, guided by WGA (human) or tomato and podocalyxin (mouse) staining. The semi-quantitative scoring for patient biopsies was performed visually for each glomerulus. The glomerular staining intensity was graded from 3 to 0, with 3 representing the strongest staining intensity and 0 representing no visible staining. In mice the same system was applied only that 30 glomeruli were randomly chosen per animal. For the mesangial index scoring, the severity of the mesangial expansion was graded with numbers from unaffected (=0), slightly expanded (=1) or moderately expanded (=2). Randomly chosen 20 glomeruli were scored from each mouse and the sum of these glomeruli represents the mesangial index.

### Mouse experiments

All animal-related methods were performed in accordance with the relevant guidelines and regulations, which were approved by the ethical Committee on Research Animal Care (Linköpings djurförsöksestiska nämd DNR41-15).

Coro2b knock-out mouse strains were generated in C57B/6J background using standard gene targeting in embryonal stem cells (Cyagen, Santa Clara, CA) by inserting loxP sites around exons 6–8 of Coro2b gene. The mice were crossed with ella-cre^11^ and podocin-cre^18^ lines to produce whole body and conditional KO mouse lines. Inactivation of Coro2b was confirmed using PCR, Western blotting and immunohistochemistry. Diabetes was induced using streptozotocin (Sigma Aldrich, Germany) as described previously^19^. Blood glucose levels were measured 12 days after the start of the experiment and at the end of the experiment. Urine was analyzed for albumin level every other week and at 5 months for the albumin/creatinine ratio with the Albuwell M assay (Exocell Inc., Philadelphia, PA) according to the manufacturer’s guidelines. Animals were sacrificed 6 months after the start of the experiment. Perfusion of 4-month old mice with protamine sulfate and heparin sulfate was performed as described before^20^. Kidneys were fixed with 2.5% glutaraldehyde and processed for standard transmission electron microscopy. Foot process effacement was quantified by calculating the number of slit diaphragms per GBM distance: three glomeruli/sample were randomly chosen and three capillaries/glomerulus were analyzed with a total of 213 ± 32 µm of GBM length analyzed per animal.

### Statistical methods

Statistical calculations were performed with excel. Depending on the underlying data we performed either an independent two-tailed distributed student’s t-test, one way ANOVA with a Tukey-Kramer post-hoc correction or a two way ANOVA with reproduction for correlation analyses.

## Discussion

Glomerular podocytes have unique morphological and functional features enabling them to form the final ultrafiltration barrier in the kidney. To accomplish this, podocytes express many genes that are found only sparsely in other cell types. In this study, we characterized Coro2b as a molecular component of podocytes. We show that: (1) Coro2b expression is highly restricted to podocyte cells where it localizes to the apical plasma membrane; (2) Coro2b is down-regulated in patients with DN; (3) Coro2b is not essential for the murine development or maintenance of the glomerular filtration barrier as podocyte specific Coro2b KO animals show no glomerular abnormalities; (4) Coro2b seems to modulate the development of foot process effacement in a mouse model as Coro2b-deficiency partially protects podocytes from PS-induced effacement. Our study suggests a potential role for Coro2b in the pathogenesis of glomerular diseases.

Proteinuria is the clinical hallmark sign of DN, and is almost invariably associated with the effacement of podocyte foot processes. We observed that glomerular expression of Coro2b is downregulated in patients with DN, suggesting that it could have a role in the development of diabetic glomerular damage. Coro2b-deficiency did not affect the development of nephropathy in the STZ-induced diabetes mouse model, which argues against the idea that Coro2b has a pathogenic role in the progression of DN. However, we speculate that the lack of phenotypical changes in KO animals may reflect on how poorly mouse models for DN phenocopy the situation in man. It is well established that most current mouse DN models lack translation to humans and therefore fail to validate findings in humans^21^. In fact, we did not detect any significant down-regulation of Coro2b expression in our mouse models of DN. The hyperglycemic extracellular condition is the main difference between DN and other glomerulopathies. It is possible that podocytes down-regulate Coro2b to protect themselves from the stress induced by abnormally high glucose levels. Hyperglycemia induces a number of different stress reactions within cells, such as ROS formation and downregulation of the AKT-signalling pathway, facilitating processes leading to cell death^3^. Down-regulation of Coro2b may stabilize podocyte foot process structure and in that way promote cell survival under diabetic stress. Evidently, more studies are needed to dissect the role of Coro2b in DN, including studies using different mouse models of DN.

Our results in the PS model suggest that Coro2b regulates the development of foot process effacement. How this occurs mechanistically was not in the scope of this study. However, it is known that during PS perfusion podocalyxin, the major apical sialoglycoprotein of podocytes, gets phosphorylated and loses its negative charge responsible for maintaining the distinct foot process architecture^22^. In this study we could show that Coro2b mainly localizes to the apical membrane of podocytes. The apical localization proposes a possible association with podocalyxin, which itself is known to interact with actin-binding proteins like ezrin^23^. We speculate that Coro2b can be a part of a molecular complex (along with ezrin) that connects podocalyxin to the actin cytoskeleton, and therefore facilitates the signal transduction between the extracellular environment and the cytoskeleton. The lack of Coro2b in the podocyte specific KOs results therefore, in a delayed/suppressed response to both PS and HS treatment. On the other hand, Coro2b has previously been shown to bind to vinculin, a scaffold protein of focal adhesions (FAs) traditionally localized basally^10,24^. FAs have an important role in the attachment of podocytes to the GBM. As we detected Coro2b mainly apically, it may be that in podocytes Coro2b does not associate with FAs. More studies are obviously needed to understand the role of Coro2b in podocytes during normal physiology and in disease processes.

While this manuscript was under preparation, Rogg *et al*. published a paper on Coro2b in human and mouse podocytes^9^. They performed detailed studies in cultured podocytes, and showed that constitutive KO mice have normal glomerular structure and function but are partially protected from doxorubicin-induced glomerular injury. In our study, we focus on *in vivo* studies and an advantage is the generation of a podocyte-specific KO mouse line. Coro2b is expressed by immune cells (www.proteinatlas.org) and inactivation of Coro2b in these cells can potentially modulate the progression of kidney disease in different injury models. However, the results in their constitutive KO mouse model are well in line with ours as they show tendencies for reno-protective effects. In summary, our study and the study by Rogg *et al*. introduce Coro2b as a new molecular component of podocytes and suggest that it has a role in the pathogenesis of glomerulopathies.

## Supplementary information

Supplemental information

## Data Availability

The manuscript contains supporting material and data, which is also shared in supplemental to enable repetition of performed and described experiments. If more detailed information about the experiment procedures is needed, we are happy to comply with sufficient information upon request.
