# Supplementary material for: Coro2b, a podocyte protein downregulated in human diabetic nephropathy, is involved in the development of protamine sulphate-induced foot process effacement
Source: Sci Rep. 2019 Jun 20;9:8888. doi: 10.1038/s41598-019-45303-y (PMC6586875; doi:10.1038/s41598-019-45303-y)
Supplement: Supplementary file 1 — Supplemental information [file 41598_2019_45303_MOESM1_ESM.pdf]

Coro2b, a podocyte protein downregulated in human diabetic nephropathy, is involved in the development of protamine sulphate-induced foot process effacement

Angelina Schwarz[1], Katja Möller-Hackbarth[1], Lwaki Ebarasi[1], David Unnersjö Jess[2], Sonia Zambrano[1], Hans Blom[2], Annika Wernerson[3], Mark Lal[4], Jaakko Patrakka[1]*

[1]Karolinska Institutet/AstraZeneca Integrated Cardio Metabolic Centre, Department of Laboratory Medicine, Karolinska Institutet at Karolinska University Hospital Huddinge, Stockholm, Sweden;

[2]Science for Life Laboratory, Dept. of Applied Physics, Royal Institute of Technology, Solna, Sweden

[3]Division of Renal Medicine, Department of Clinical Sciences, Intervention and Technology, Karolinska Institutet, Stockholm, Sweden

[4]Bioscience, Cardiovascular, Renal and Metabolism, Innovative Medicines Biotech Unit, AstraZeneca, Gothenburg, Sweden

<u>Correspondence:</u>

Jaakko Patrakka MD PhD

Karolinska Institutet/AstraZeneca Integrated Cardio Metabolic Centre

Karolinska Institutet at Karolinska University Hospital Huddinge

14186 Huddinge

Sweden

jaakko.patrakka@ki.se

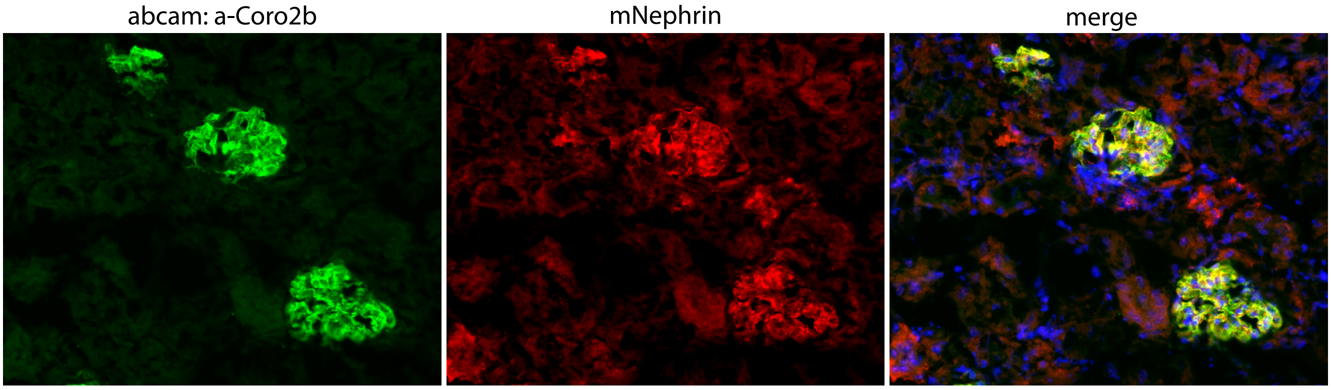

Suppl. figure 1: Immunofluorescent staining of murine kidney with alternative anti-Coro2b antibody. Co-stainng of murine kidney sections with alternative antibody targeting Coro2b (green) and anti-nephrin (red) shows similar glomerular reactivity as the first anti-Coro2b antibody. Magnifications: 40x.

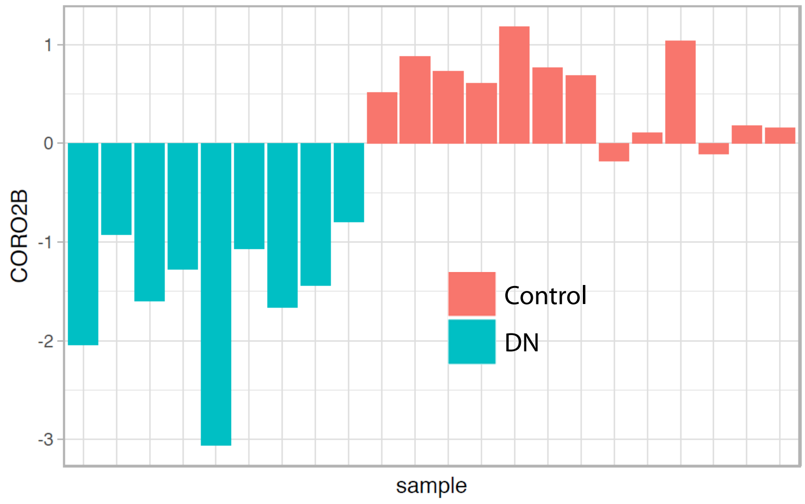

Suppl. figure 2: Microarray data of Coro2b's expression levels in DN and control glomeruli. The bar graph shows the expression levels of Coro2b within isolated glomeruli from DN patients (blue) and control patients (red). Each column represents a patient. The data from Woroniecka *et. al.* support the downregulation of Coro2b in DN.

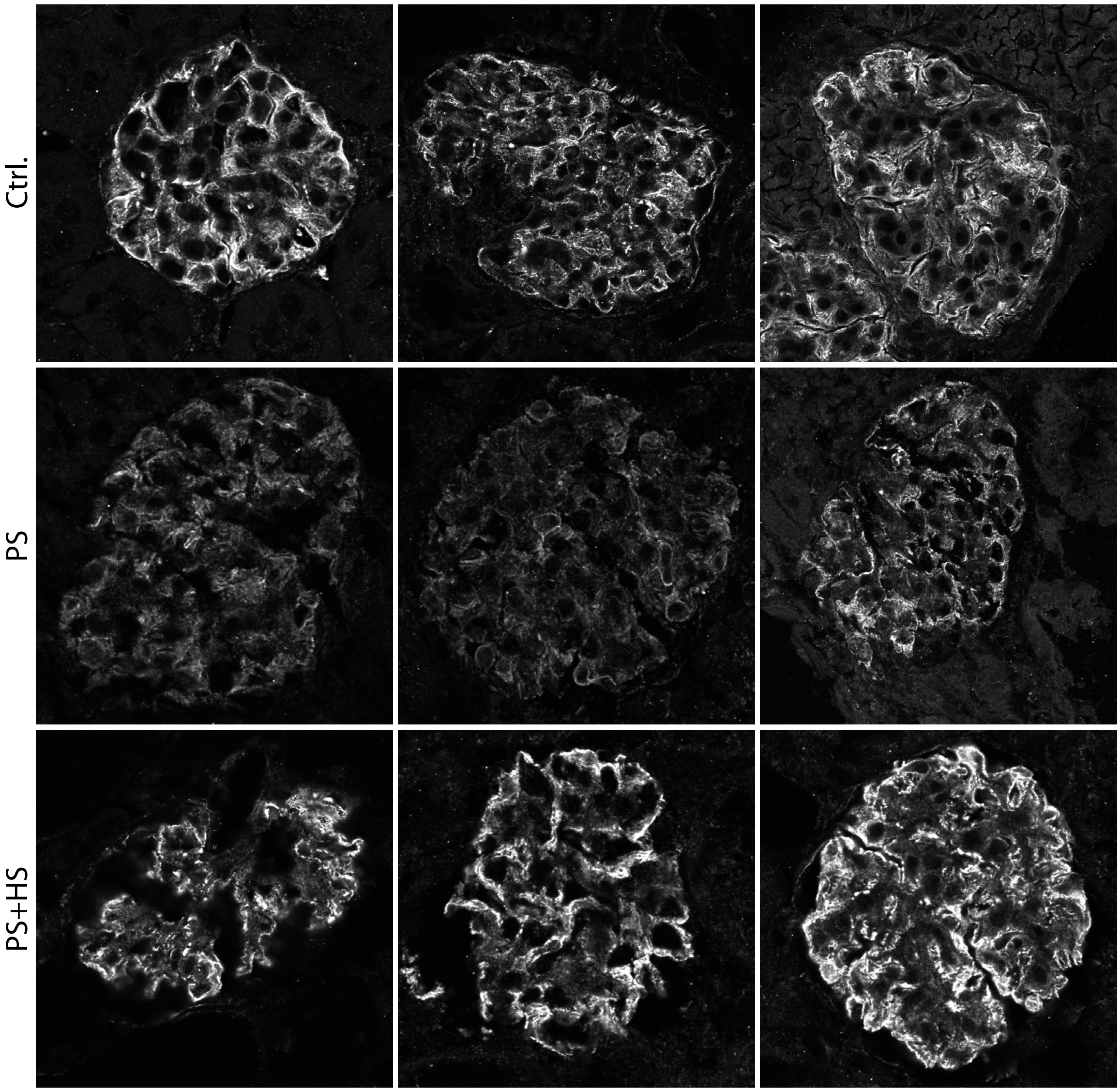

Suppl. figure 3: Immunofluorescent anti-Coro2b staining of PS and PS+HS treated WT animals. The immunofluorescent stainings showed a reduction/redistribution of Coro2b signal of PS perfused WT glomeruli compared to the HS rescued (PS+HS) WT or Ctrl. glomeruli, indicating this stress model affects Coro2b. Magnifications: 40x.

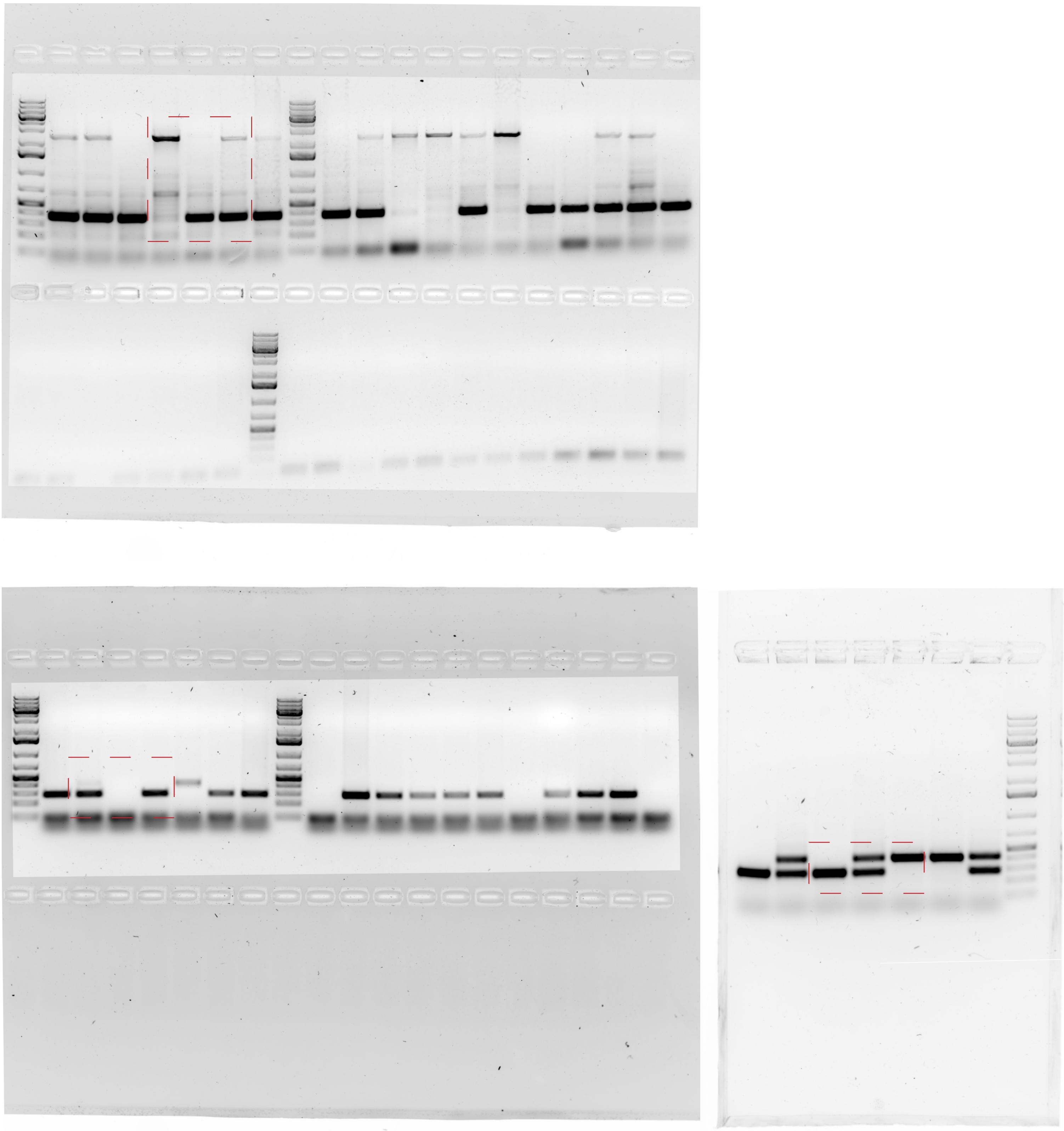

Suppl. figure 4: Uncropped gel pictures from genotyping in figure 4. Upper gel is for the “genome”-primers Lower two gels are for the “casette” primers. Dashed lines indicate the crop-out for figure 4.

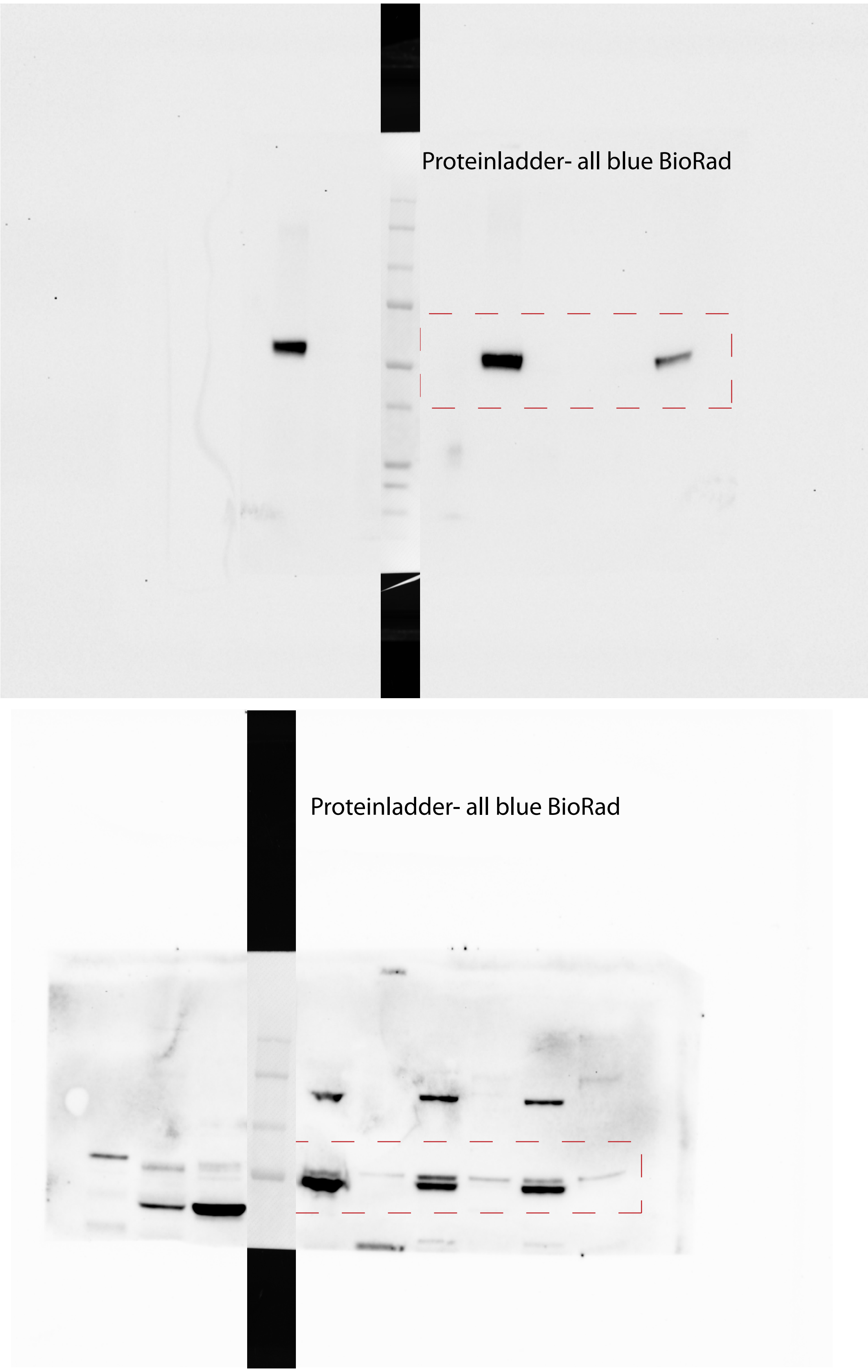

Suppl. figure 5: Uncropped blot pictures for Western Blots in figure 4. Upper blot is a-Coro2b and lower blot is a-Calnexin. Lower membrane was cut in two for using it with an other antibody (which did not work properly). Both pictures are from the same mebrane, a-calnexin was used after strippping away a-Coro2b. Dashed lines indicate the crop-out for figure 4.

Supplemental Table 1. Parameters at the time of biopsy for DN, IgAN and MN patients included in immunohistochemical studies. Control samples were from nephrectomized kidneys. NA = not available.

Histological diagnosis	Age	Gender	P-Crea (µmol/mol)	eGFR	U-Alb (mg/L)
DN	50	Male	626	9	3436
DN	68	Male	367	15	16
DN	55	Male	131	52	NA[1]
DN	54	Female	161	31	NA[2]
DN	69	Male	165	38	112
DN	68	Male	80	89	NA[3]
DN	67	Male	130	51	1212
DN	62	Male	78	93	654
DN	63	Male	194	32	93
IgAN	33	Male	106	74	27
IgAN	21	Female	52	137	380
IgAN	37	Male	132	56	6
IgAN	50	Male	143	48	15
IgAN	26	Male	84	102	50
IgAN	43	Male	372	17	543
IgAN	46	Male	277	23	1390
IgAN	64	Male	559	10	2828
IgAN	66	Male	586	9	1317
IgAN	62	Male	220	28	702
MN	53	Male	73	104	616
MN	41	Male	79	100	7635
MN	60	Female	75	73	13400
MN	69	Male	193	32	7943
MN	51	Male	75	101	1904
Control	48	Male	96	77	NA
Control	58	Male	84	87	NA
Control	59	Male	83	87	NA

[1]U-Albumin/creatinine 328 mg/mmol
[2]U-Albumin/creatinine 470 mg/mmol
[3]U-Albumin/creatinine 276 mg/mmol

Supplemental table 2: Correlation of individual patient Coro2b staining intensities with their correlating GFR value.

			Intensity - Coro2b [%]			
**Diagnosis**	**GFR**	**CKD**	**3**	**2**	**1**	**0**
MN	104	1	90	10	0	0
MN	100	1	71	29	0	0
MN	73	2	27	73	0	0
MN	32	3	100	0	0	0
MN	101	1	100	0	0	0
DN	38	3	0	0	75	25
DN	89	2	0	0	43	57
DN	51	3	0	6	38	56
DN	93	1	0	18	45	36
DN	32	3	0	30	40	30
DN	15	5	0	0	30	70
DN	31	3	0	6	31	63
DN	52	3	0	38	38	23
DN	9	5	0	13	22	65
IgAN	102	1	24	52	24	0
IgAN	48	3	100	0	0	0
IgAN	56	3	0	67	33	0
IgAN	137	1	0	0	0	100
IgAN	74	2	27	55	18	0
IgAN	10	5	28	44	28	0
IgAN	28	4	0	50	50	0
IgAN	9	5	90	10	0	0
IgAN	10	5	8	60	32	0
IgAN	23	4	80	20	0	0

Supplemental table 3: Primer sequences

	**Primer**	**Sequence: 5' -> 3'**
**Human**	hCoro2b-F	AATGGAACCCCTTCATCGAC
	hCoro2b-R	AGTTGGCCTCCTGCAGAACA
	28S-F	TTGAAAATCCGGGGGAGAG
	28S-R	ACATTGTTCCAACATGCCAG
**MOUSE**	mCoro2b-F	GAGCTCCACGGACACAGC
	mCoro2b-R	GGCGATCTGTCTTGTGTTCC
	mCoro2b-geno1-F	GTGGCTCTGAAGCCAGGTCTATAGC
	mCoro2b-geno-KO-F	ACCAAATTCCAGACTTGTTCTTGCTTC
	mCoro2b-geno-R	GAGGGCTCTAGGTCTTTCCAACTGG
	mGAPDH-F	TGTTCCTACCCCCAATGTGT
	mGAPDH-R	TGTGAGGGAGATGCTCAGT

Supplemental table 4: Antibodies

**Antibody**	**Host**	**Against**	**Company**	**Catalouge**
Calnexin	rabbit	human	abcam	ab10286
CD31	mouse	human	abcam	ab24590
Coro2b	rabbit	human, mouse	atlas antibodies	hpa017960
Coro2b	rabbit	human, mouse	abcam	ab151327
Hoechst 33342	-	DNA	ThermoFischer scientific	H21492
Nephrin	mouse	human	Karolinska	50HA
Nephrin	sheep	human, mouse	RnD systems	AF4269
PDGFRbeta	mouse	human	RnD systems	MAB1263
Podocalyxin	mouse	human	RnD systems	FAB1658G
Podocalyxin	goat	mouse	RnD systems	AF1556
Synaptopodin	mouse	human, mouse	Progen	61094
Wheat Germ Agglutinin	wheat	sialic acid and N-acetylglucosaminyl residues	Sigma-Aldrich	L9640-10MG
